# Anti-Müllerian Hormone: A Valuable Addition to the Toolbox of the Pediatric Endocrinologist

**DOI:** 10.1155/2013/674105

**Published:** 2013-12-08

**Authors:** Nathalie Josso, Rodolfo A. Rey, Jean-Yves Picard

**Affiliations:** ^1^INSERM U782, Université Paris-Sud, UMR-S0782, 92140 Clamart, France; ^2^Centro de Investigaciones Endocrinológicas “Dr. César Bergadá” (CEDIE), CONICET-FEI-División de Endocrinología, Hospital de Niños “R. Gutiérrez”, C1425EFD Buenos Aires, Argentina; ^3^Departamento de Histología, Embriología, Biología Celular y Genética, Facultad de Medicina, Universidad de Buenos Aires, C1121ABG Buenos Aires, Argentina

## Abstract

Anti-Müllerian hormone (AMH), secreted by immature Sertoli cells, provokes the regression of male fetal Müllerian ducts. FSH stimulates AMH production; during puberty, AMH is downregulated by intratesticular testosterone and meiotic germ cells. In boys, AMH determination is useful in the clinical setting. Serum AMH, which is low in infants with congenital central hypogonadism, increases with FSH treatment. AMH is also low in patients with primary hypogonadism, for instance in Down syndrome, from early postnatal life and in Klinefelter syndrome from midpuberty. In boys with nonpalpable gonads, AMH determination, without the need for a stimulation test, is useful to distinguish between bilaterally abdominal gonads and anorchism. In patients with disorders of sex development (DSD), serum AMH determination helps as a first line test to orientate the etiologic diagnosis: low AMH is indicative of dysgenetic DSD whereas normal AMH is suggestive of androgen synthesis or action defects. Finally, in patients with persistent Müllerian duct syndrome (PMDS), undetectable serum AMH drives the genetic search to mutations in the AMH gene, whereas normal or high AMH is indicative of an end organ defect due to AMH receptor gene defects.

## 1. Introduction

Anti-Müllerian hormone (AMH), also known as Müllerian inhibiting substance (MIS) or factor (MIF), is a member of the transforming growth factor-*β* (TGF-*β*) secreted essentially by fetal and prepubertal Sertoli cells and to a lesser amount by granulosa cells of small follicles. AMH plays a biological major role in shaping the male reproductive tract by triggering the regression of male fetal Müllerian ducts while androgens, secreted by the Leydig cells present in the interstitial tissue, are responsible for the stabilization of the Wolffian ducts and their differentiation into male accessory organs as well as for the virilization of the urogenital sinus and the external genitalia. In males lacking AMH, the persistence of Müllerian derivatives coexists with the development of normal male external genitalia. It follows that clinical applications of AMH in pediatric endocrinology are essentially diagnostic and restricted to boys. In recent years, AMH has gained great importance in gynecology and obstetrics, due to its value as a marker of ovarian reserve but this clinical application does not concern pediatricians and will not be considered here. Several AMH ELISA kits are commercially available as discussed elsewhere in this issue.

## 2. Ontogeny and Regulation of Testicular AMH Production

AMH is a homodimeric glycoprotein member of the TGF-*β* family. It is initially secreted as a precursor, subsequently cleaved to yield 110 kDa N-terminal and 25-kDa C-terminal homodimers, which remain associated as a biologically active noncovalent complex [1]. Dissociation of the noncovalent complex occurs at the time of binding to type II AMH receptor and is required for biological activity [2]. The major site of AMH production is the immature Sertoli cell. In the late fetal and postnatal ovary, it is also produced by granulosa cells of developing follicles, essentially preantral and small antral follicles [3, 4].

**Figure 1:**
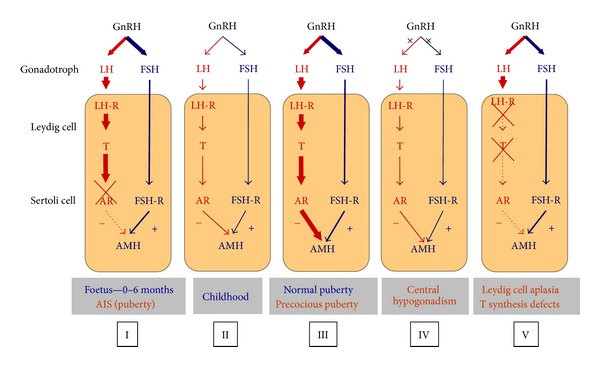
Regulation of testicular AMH secretion by gonadotropins and androgens. In general, the hypothalamus regulates LH and FSH secretion by the gonadotroph through the gonadotropin-releasing hormone (GnRH). LH acts on the LH receptor (LH-R) present in Leydig cells, inducing testosterone (T) secretion. FSH acts on the FSH receptor (FSH-R) present in Sertoli cells. The hypothalamic-pituitary-gonadal axis is active in the foetus and early infancy, is quiescent during childhood, and is reactivated at puberty. FSH is a moderate inducer of AMH secretion, whereas T, acting through the androgen receptor (AR), is a potent inhibitor of AMH production. In the normal foetus and infant, as well as in patients with the androgen insensitivity syndrome (AIS), the lack of AR expression results in high AMH production by Sertoli cells (I). During childhood, there is a physiologic hypogonadotropic state resulting in very low T; AMH levels remain high, but somewhat lower probably due to the lack of FSH stimulus (II). In normal or precocious puberty, T prevails over FSH, resulting in AMH inhibition (III). In congenital central hypogonadism, AMH is lower than in the normal boy because of the longstanding lack of FSH from foetal life; however, at pubertal age, the inhibitory effect of T is also absent, and AMH remains higher than in normal puberty (IV). In Leydig cell-specific primary hypogonadism (Leydig cell aplasia or hypoplasia due to LH-R defects or defects of steroidogenesis), the inhibitory effect of androgens is absent, and AMH levels are high. The orange area represents the testis. Thickness of lines is in correlation with hormone effect on its target. From [5], Copyright Karger AG, 2010, with permission.

In the male, AMH is a specific functional marker of the immature Sertoli cell. AMH expression is initiated at the time of fetal differentiation of the seminiferous cords, by the end of the 7th embryonic week, and remains at high levels until the onset of puberty, except for a transient decline in the perinatal period [6, 7]. AMH expression is triggered by SOX9, which binds to the AMH promoter; subsequently, SF1, GATA4, and WT1 further increase AMH promoter activity (reviewed in [8]). The onset of AMH expression and its basal expression level throughout life are independent of gonadotropins. However, FSH stimulates testicular AMH production by both inducing Sertoli cell proliferation and upregulating AMH transcription [9]. The latter is mainly mediated by the classical pathway involving Gs*α* and adenylyl cyclase increase of cyclic AMP levels, which stimulates protein kinase A (PKA) activity, subsequently involving transcription factors SOX9, SF1, GATA4, NF*κ*B, and AP2 [10–12]. During puberty, AMH production is inhibited by the increase of intratesticular testosterone concentration and the onset of germ cell meiosis (Figure 1) (reviewed in [13, 14]). The inhibitory effect of androgens on AMH overcomes the positive effect of FSH after pubertal onset. On the contrary, androgens cannot inhibit AMH production in the fetal and neonatal testis, where Sertoli cells do not yet express the androgen receptor [15–17].

## 3. AMH in Boys with Hypogonadism

Gonadotropin and testosterone, which are high in the 3–6 months after birth, normally decrease to very low levels until the onset of puberty. Therefore, their usefulness as markers of the function of the hypothalamo-pituitary-gonadal axis in the boy is limited. On the contrary, AMH determination is extremely useful, since Sertoli cells remain active during infancy and childhood [18, 19]. Serum AMH reliably reflects the presence and function of testes in prepubertal boys, without the need for any stimulation test [18, 20, 21]. In this section, we address how the different disorders causing hypogonadism affect AMH testicular production.

### 3.1. Central Hypogonadism

**Figure 2:**
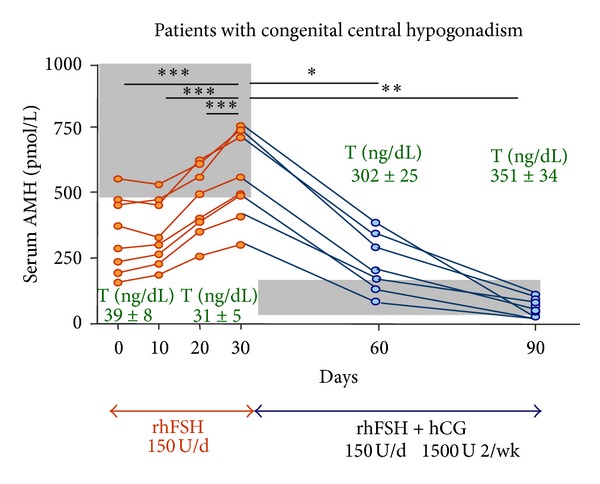
AMH levels in central hypogonadism. Serum AMH was low for Tanner stage I (prepubertal) in patients with previously untreated central hypogonadism. Initial treatment with recombinant human FSH (rhFSH) during 30 days resulted in an elevation of serum AMH in all 8 patients, while testosterone (T) remained at prepubertal levels. Shaded area represents normal AMH for Tanner I stage, according to T levels observed in these patients. Subsequent addition of hCG treatment resulted in an elevation of T which provoked a decline in serum AMH. Shaded area represents AMH values for Tanner IV-V stages, according to T levels observed in the treated patients. From [5], Copyright Karger AG, 2010, with permission.

Serum AMH is low in infants with congenital central hypogonadism. Treatment with FSH results in an elevation in serum AMH in correlation with an increase in testis volume [22]. In patients of pubertal age with untreated congenital central hypogonadism, serum AMH is elevated for age—because the insufficient testosterone production is unable to downregulate AMH, but lower than expected for patient's Tanner stage [23, 24]—reflecting the lack of FSH stimulus. FSH treatment results in an increase in serum AMH; subsequent treatment with hCG induces androgen production, which provokes a physiological decline in AMH (Figure 2). Interestingly, inhibition of AMH does not occur when patients are treated with exogenous testosterone, which reflects that intratesticular testosterone levels remain low [24].

### 3.2. Primary Hypogonadism

In patients with sex-chromosome aneuploidies resulting in Klinefelter syndrome (47,XXY), no overt signs of hypogonadism are evident during infancy and childhood: AMH, inhibin B, and FSH levels are normal. However, from midpuberty Sertoli cell function deteriorates progressively, resulting in extremely low or undetectable AMH and inhibin B levels, very high FSH, and small testis volume [25].

Unlike Klinefelter syndrome, the somatic aneuploidy of Trisomy 21 (Down syndrome) results in early-onset primary hypogonadism in a large proportion of cases. Serum AMH is low from infancy [22].

Patients with Prader-Willi syndrome have hypogonadism leading to small genitalia and arrested pubertal development, classically attributed to hypothalamic dysfunction. However, recent investigations have demonstrated that the disorder may also be due to primary hypogonadism, with low AMH and testosterone levels associated with normal to moderately elevated gonadotropins [26–28] or to a combined form of hypogonadism, with low testicular hormones and inadequately normal gonadotropins [27, 29].

The X-linked form of adrenal hypoplasia congenita associated with hypogonadism resulting from mutations in the DAX1 gene is another example of combined (central + primary) hypogonadism. These patients have low serum AMH and inhibin B and defective androgen response to hCG, indicative of a primary testicular failure. At pubertal age, gonadotropin levels remain inadequately normal in spite of the lack of negative feedback resulting from low inhibin B and testosterone, which indicates that gonadotrope function is also impaired [30].

### 3.3. Cryptorchidism

**Figure 3:**
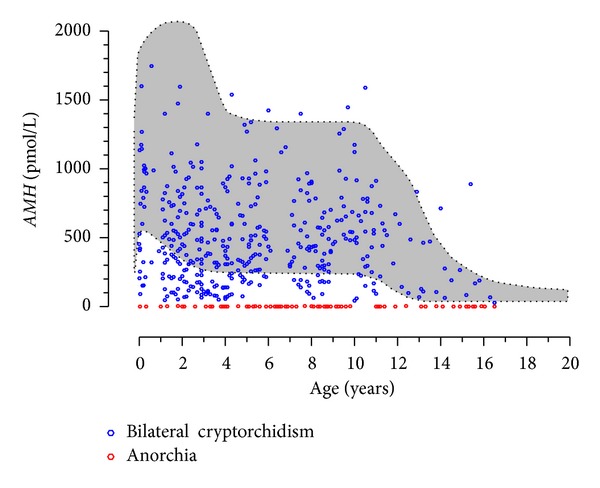
Serum AMH levels are useful to distinguish between bilateral cryptorchidism with abdominal testes and anorchism. Serum AMH is undetectable in anorchid patients; in patients with bilaterally abdominal testes, serum AMH is always detectable ranging from subnormal to normal values according to the functional status of the gonads. Shaded area represents the normal serum AMH range (3th–97th centiles), according to [22].

Cryptorchidism is a sign that can be present in many disorders of different etiologies, most of which remain elusive [31, 32]. Dissociated testicular dysfunction primarily affecting the tubular compartment seems to be the underlying pathophysiology in cases presenting with low AMH [33] and inhibin B [34] but with normal testosterone and INSL3 [35] during early infancy and childhood. In other cases, no significant changes in hormone levels could be detected [36]. The apparently contradictory results are most probably due to the heterogeneity of the cryptorchid patients with underlying conditions of different etiologies and prognoses. Bilateral cryptorchidism with nonpalpable gonads should be distinguished from anorchia. Vanishing or regression of testicular tissue occurring in late fetal life, once sex differentiation has occurred, is associated with male genitalia, micropenis, and hypoplastic scrotum. Later in postnatal life, anorchia should be distinguished from bilateral cryptorchidism with abdominal testes. Serum AMH is undetectable in anorchid boys but detectable in boys with abdominal gonads (Figure 3) [20, 21, 37].

In Noonan syndrome, cryptorchidism occurs in approximately 2/3 of the cases. During childhood, reproductive hormones are within the expected range. Pubertal onset is delayed; by mid- to late puberty, gonadotropin levels increase over the normal range and AMH and inhibin B decline to subnormal levels in patients with a history of cryptorchidism but remain within normal levels in those with descended testes [38].

## 4. AMH in Disorders of Sexual Differentiation

The development and differentiation of the sex organs during fetal life involve three successive steps: (1) the early morphogenesis of the gonadal and genital primordia, which is identical in XY and XX embryos; (2) the differentiation of the gonadal ridge into a testis or an ovary; (3) the differentiation of the primordia of the internal and external genitalia, which are virilized by the action of androgens and AMH or feminized in their absence.

Based on the recognition of the cause of abnormal sex organ development in patients bearing a Y chromosome (Table 1), disorders of sex development (DSD) may be divided into (a) malformative DSD, where abnormal morphogenesis of the genital primordia occurs in early embryonic life; (b) dysgenetic DSD, due to abnormal gonadal differentiation resulting in insufficient secretion of androgens and AMH; and (c) nondysgenetic DSD, in which the abnormal sex hormone-dependent genital differentiation results from specific defects in the production or action of androgens or AMH.

### 4.1. AMH in Malformative DSD

Defects in the early morphogenesis of the Müllerian or Wolffian ducts, the urogenital sinus, or the primordia of the external genitalia, for example, cloacal malformations, isolated hypospadias, or aphallia, usually occur in eugonadal patients. Therefore, serum AMH and testosterone levels are within the expected range for sex and age. From a practical standpoint, nonendocrine related DSD should be considered when there is inconsistency in the development of the different elements of the genitalia. For instance, isolated hypospadias, with no other signs of hypovirilization, in patients with normal AMH and androgen levels is most probably due to early morphogenetic defects [39, 40]. In most cases, endocrine-unrelated malformations of the genitalia are associated with other somatic dysmorphic features, like in Robinow syndrome due to *ROR2* mutations, Pallister-Hall syndrome due to *GLI3* mutations, or many other polymalformative associations of unknown etiology. “Idiopathic” persistence of Müllerian derivatives (PMDS) in patients with a normal AMH level, mutation-free AMH, and AMH receptor genes may belong to the same category (see below).

### 4.2. AMH in Dysgenetic DSD

**Figure 4:**
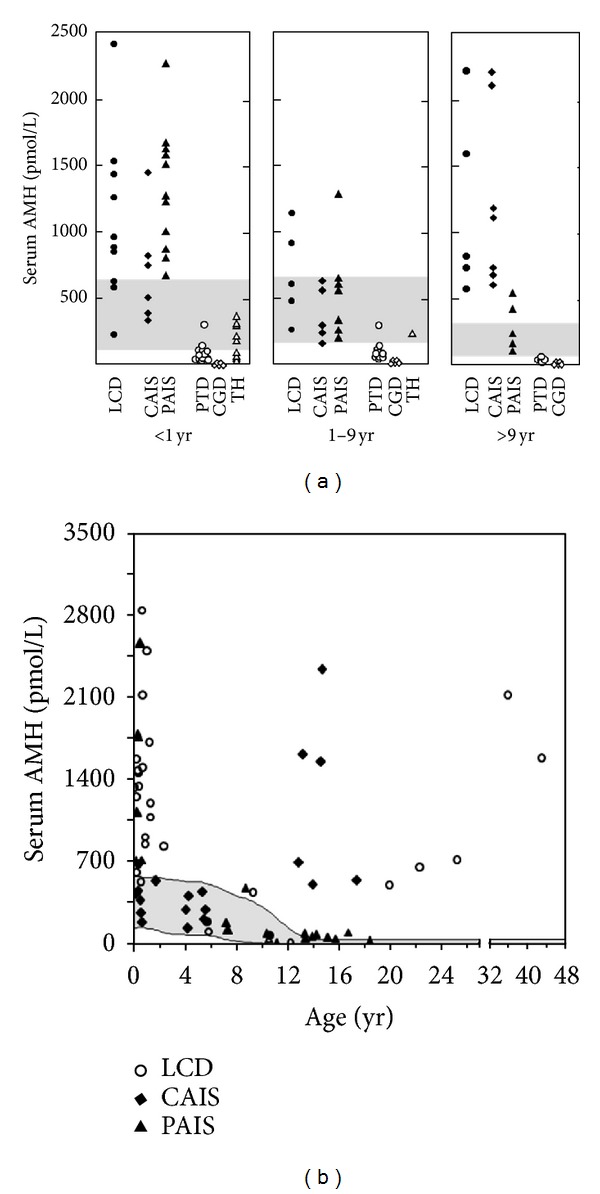
Serum AMH in disorders of sex development (DSD). (a) Serum AMH levels in patients with DSD. LCD: Leydig cell defects, including Leydig cell aplasia or hypoplasia and steroidogenic enzyme mutations; CAIS: complete androgen insensitivity syndrome; PAIS: partial androgen insensitivity syndrome; PTD: partial testicular dysgenesis, including asymmetrical gonadal differentiation; CGD: complete gonadal dysgenesis; TH: true hermaphroditism or ovotesticular DSD. The shaded areas represent the normal levels. Data is obtained from [41]. Copyright, The Endocrine Society, 1999. (b) Serum AMH levels in patients with DSD due to defects in androgen production (Leydig cell defects, LCD) or action (complete or partial androgen insensitivity syndrome, AIS). The shaded area represents the normal levels. Data is obtained from [44]. Copyright, The Endocrine Society, 1994, with permission.

Gonadal dysgenesis established in the first trimester of fetal life represents the earliest form of primary hypogonadism and prevents the normal hormone-driven differentiation of the sex organs. In the fetus carrying a Y chromosome, gonadal dysgenesis results in female or ambiguous genitalia, reflecting the degree of testicular hormone deficiency. Serum AMH is low or undetectable, depending on the amount of testicular tissue remaining [41] (Table 1 and Figure 4). Serum AMH observed in a newborn with ambiguous genitalia should be compared with reference levels for the adequate age period to avoid overdiagnosis of dysgenetic DSD. AMH levels are transiently lower during the first 2-3 weeks after birth in the normal newborn [6, 7, 22]; when in doubt, a repeat measurement to assess the evolution of serum AMH may be helpful [42].

In 45,X or 45,X/46,XX patients, gonads are reduced to fibrous streaks or develop into dysgenetic ovaries. Serum AMH levels reflect the amount of small follicles present in these gonads and predict the occurrence of spontaneous pubertal onset [43].

Ovotesticular DSD is a particular type of gonadal dysgenesis where both testicular and ovarian tissues are present. The most frequent karyotypes are 46,XX or mosaicism including at least one XY lineage. The degree of virilization is usually commensurate with the amount of testicular tissue. In XX patients, the differential diagnoses are congenital adrenal hyperplasia, aromatase deficiency, and androgen-secreting tumors. An increased level of serum AMH is specific of ovotesticular DSD [41]; in the other conditions serum AMH is in the female range. In contrast, androgen assay is not useful for diagnosis, since androgens are always above normal female levels.

### 4.3. AMH in DSD due to Defects in Androgen Synthesis or Action

While gonadal dysgenesis affects the production of both androgens and AMH, DSD may also result from a specific defect impairing the endocrine function of Leydig cells. In this case, there is a “dissociated” or “cell-specific” form of fetal-onset primary hypogonadism (reviewed in [5]), as opposed to gonadal dysgenesis leading to whole gonadal failure. Deficiency of androgen synthesis results in the occurrence of female or ambiguous external genitalia and no uterus.

#### 4.3.1. Leydig Cell Aplasia/Hypoplasia and Steroidogenic Protein Defects

Leydig cell aplasia, due to inactivating mutations of the LH/CG receptor, and defects in proteins or enzymes involved in testicular steroidogenesis result in complete lack or insufficiency of androgen production by the testes. Consequently, hypovirilization or feminization of genitalia occurs as in dysgenetic DSD. Both dissociated primary hypogonadism specifically affecting Leydig cells and dysgenetic DSD have low testosterone levels in serum, yet it is possible to distinguish them by measuring AMH. While AMH is low or undetectable in dysgenetic DSD, as described above, it is normal/high in steroidogenic defects because the androgen inhibitory effect on AMH is lacking and the elevation of serum FSH upregulates AMH secretion [41], particularly in the first 3–6 months after birth and at pubertal age in those cases where gonadectomy has not yet been performed (Table 1 and Figure 4). It should be noted that AMH may be within the normal male range in these patients during childhood.

#### 4.3.2. Deficiency of 5*α*-Reductase

Steroid 5*α*-reductase is the key enzyme for the conversion of testosterone to dihydrotestosterone (DHT). The androgen receptor has a higher affinity for DHT than for testosterone. In the absence of 5*α*-reductase activity, the Wolffian ducts differentiate normally because the adjacent testes supply sufficiently high local testosterone concentrations. Conversely, more distant androgen-dependent organs, like the urogenital sinus and the external genitalia, need testosterone conversion to DHT for adequate virilization. The Müllerian ducts regress normally because Sertoli cell AMH production is not affected. Testosterone levels are normal, and serum AMH is also within the normal male range. Because there are normal testicular androgen concentration and androgen receptor expression and FSH is not elevated, serum AMH is not increased in these patients [45] (Table 1).

#### 4.3.3. Androgen Insensitivity Syndrome (AIS)

Androgen insensitivity due to mutations in the androgen receptor is the most frequent cause of lack of virilization in eugonadal XY patients. The testes differentiate normally, and both Sertoli and Leydig cells are functionally normal from an endocrine standpoint. Owing to end-organ insensitivity to androgens, Wolffian ducts regress, and the urogenital sinus and the external genitalia fail to virilize. Müllerian ducts do not develop, reflecting normal AMH activity. Complete AIS results in a female external phenotype, whereas partial AIS presents with ambiguous genitalia.

The pituitary-gonadal axis shows different features during the first three months of life in complete and partial AIS. In the newborn with complete AIS, FSH remains low, which probably explains why serum AMH is not as high as expected [46]. Conversely, in partial AIS, gonadotropins as well as AMH are elevated (Table 1 and Figure 4) [44, 46]. As in DSD due to defects of steroid synthesis, serum AMH remains within the normal male range during childhood [41]. At pubertal age, provided gonadectomy has not been performed, a difference is again observed between partial and complete AIS. In complete AIS, serum AMH increases to abnormally high levels, whereas in partial AIS the elevation of intratesticular testosterone concentration is capable of inducing an incomplete inhibition of AMH expression. Nonetheless, AMH levels are inadequately high for the concomitant circulating testosterone [41].

### 4.4. AMH in the Persistent Müllerian Duct Syndrome (PMDS)

PMDS is characterized by the persistence of Müllerian duct derivatives, uterus, Fallopian tubes, and upper vagina, in otherwise normally virilized 46,XY males. Approximately 85% of cases are due to mutations of the AMH or AMHR-II gene; in roughly equal proportions, 15% are idiopathic. All the information provided is current up to May 2013.

#### 4.4.1. AMH Deficiency: PMDS due to AMH Gene Mutations

*Clinical and Anatomical Features.* Because of their normal external male phenotype, patients are assigned at birth to the male gender without hesitation, in spite of the fact that one or both testes are not palpable in the scrotum. When cryptorchidism is unilateral, the contralateral scrotal sac contains a hernia, in addition to the testis. Preoperative diagnosis of PMDS is best reached by laparoscopy [47, 48]. However, unless an elder brother has been diagnosed with the condition, persistence of Müllerian derivatives is usually discovered unexpectedly during a surgical procedure for cryptorchidism and/or hernia repair.

Testes and the vasa deferentia adhere to the walls of uterus and vagina [49]. Their location depends upon the mobility of the Müllerian structures. Often, the broad ligament which anchors the uterus to the pelvis is abnormally thin, allowing the Müllerian derivatives to follow one testis through the inguinal canal and into the scrotum, resulting in “*hernia uteri inguinalis*.” The testis on the opposite side may already be present in the same hemiscrotum, a condition known as “*transverse testicular ectopia*;” this rare condition is associated with PMDS in 30% of cases [50]. Very rarely, transverse testicular ectopia is the only anatomical abnormality observed in patients homozygous for an AMH or AMHR-II mutation; no Müllerian derivatives can be detected [51].

The PMDS testis is only loosely anchored to the bottom of the processus vaginalis; the gubernaculum is long and thin, resembling the round ligament of the uterus and exposing the mobile testis to an increased risk of torsion [52] and subsequent degeneration [53]. Alternatively, the Müllerian derivatives may remain anchored in the pelvis, preventing testicular descent [54] and giving rise to bilateral cryptorchidism. The presence of these midline structures may be missed if cure is attempted through inguinal incisions. The apparent rise in the incidence of PMDS over recent years may be due to the increased use of laparoscopy in patients presenting with bilateral impalpable testes.

*Prognosis.* Pubertal development is normal; however, incontrovertible evidence of paternity is lacking. Infertility may result from aplasia of the epididymis or germ cell degeneration due to long standing cryptorchidism. However, excising the uterus to allow abdominal testes to descend into the scrotum carries significant risks to testicular blood supply. Most authors recommend partial hysterectomy, limited to the fundus and proximal Fallopian tubes or the simple division of Müllerian structures in the midline. Later, in the case of ejaculatory duct defects, intracytoplasmic sperm injection may be helpful. Orchiectomy is required if the testis cannot be brought down because of a 15% risk of cancer, an incidence apparently not higher than that for other abdominal undescended testes (reviewed in [55, 56]). AMH mutations are asymptomatic in young girls.

*Biological Features.* Testosterone and gonadotropin levels are normal for age. Serum AMH levels are generally very low or undetectable in prepubertal patients [57] due to instability of the mutant protein. This is not restricted to mutations coding for the bioactive C-terminus [58]: a 3D model of the C-terminus has been generated, using BMP2 and BMP7 as templates, providing insights into the impact of 3′ mutations upon secretion and action. One single mutation suspected of disturbing the interaction of the molecule with its type I receptor, ALK3, coexisted with a normal serum AMH concentration [58]. Thus, a normal serum AMH, albeit very rare, does not absolutely rule out the possibility of a pathogenic AMH gene mutation; however, this hypothesis cannot be entertained unless the AMHR-II gene has been totally exonerated.

**Figure 5:**
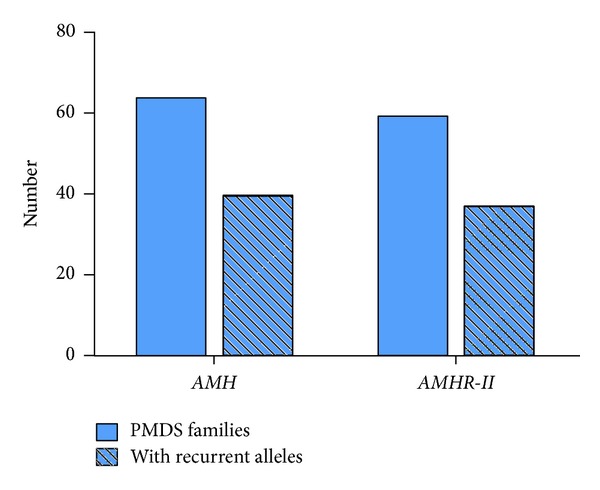
Recurrent alleles in families with persistent Müllerian duct syndrome (PMDS). Number of PMDS families and number of families with recurrent alleles. PMDS: persistent Müllerian duct syndrome; *AMH*: anti-Müllerian hormone gene; *AMHR-II*: anti-Müllerian hormone receptor type II gene.

**Figure 6:**
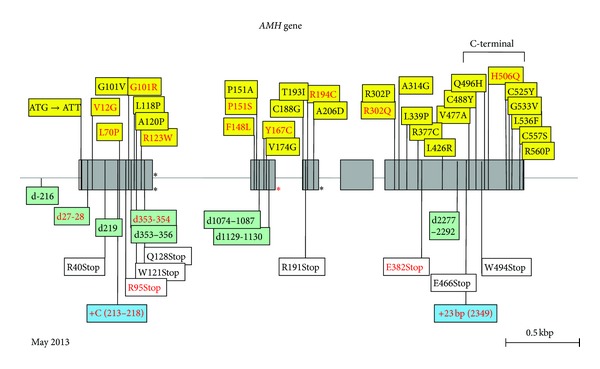
Mutations of the *AMH* gene in PMDS. Exons are shaded. All recurrent mutations are indicated in red. Missense mutations are in yellow boxes; note that the first mutation destroys the translation initiation site. Asterisks represent splice mutations; the red asterisk at the beginning of the second intron indicates a mutation detected in three different families all from Northern Europe. Deletions (marked “d”) are in green boxes, insertions (marked “+”) are in blue boxes, and nonsense mutations are in white boxes. A deletion mutation in the promoter is shown. C-terminal: coding for bioactive C-terminal domain of the AMH molecule. Base numbering is from major transcription initiation site, −10 bp from A of ATG.

**Figure 7:**
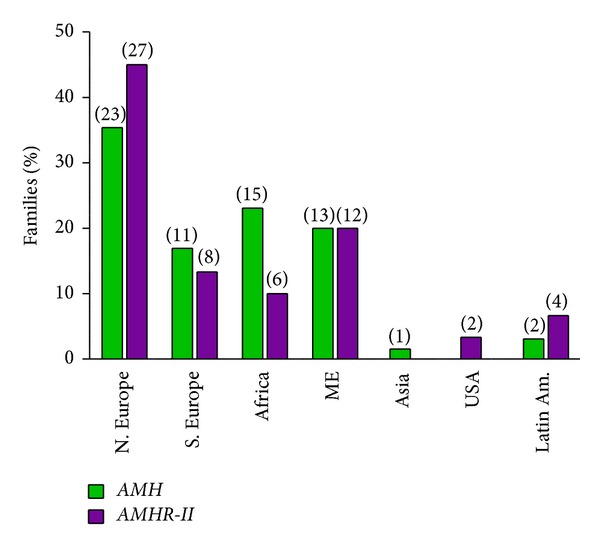
Ethnic origin of PMDS families. Results are expressed as percentages of total number of families with, respectively, AMH or AMHR-II mutations. The number of families is shown between parentheses. Differences between *AMH* and *AMHR-II* mutations are not statistically significant; the predominance of Northern Europe merely reflects a recruitment bias. N. Europe: Northern Europe (including Northern France), S. Europe: Southern Europe (including Southern France), Africa: (mostly Maghreb), ME: Middle East (includes Turkey, Afghanistan, and Pakistan, as per Wikipedia definition), Latin Am.: Latin America (includes Mexico, Central and South America).

*Molecular Genetics.* The human AMH gene, first cloned in 1986 [59], contains 5 exons. The 3′ end of the last one is extremely GC rich and shows homology to other members of the TGF-ß family; it codes for the bioactive C-terminal domain of the AMH molecule. The gene is located on the short arm of chromosome 19 [60]. PMDS is usually transmitted as an autosomal recessive trait; AMH mutations are responsible for 52% of the PMDS cases in which genetic defects have been detected. The first reported AMH mutation, a nonsense mutation of the 5th exon, was discovered in 1991 in a Moroccan family [61]. At the time of writing, May 2013, 65 families with AMH mutations (Figure 5), representing a total of 54 different alleles, have been identified (Figure 6). Except for exon 4, all exons coding both the inactive N-terminal proregion and the bioactive C-terminal mature protein are affected. All types of mutations are represented; 63% are homozygous. There is no true hotspot, though 17 abnormal alleles have been detected in more than one family. The ethnic origin of patients with documented AMH mutations is shown in Figure 7. The high proportion of European families is certainly due to a recruitment bias.

#### 4.4.2. Insensitivity to AMH: PMDS due to AMH Receptor Mutations

**Figure 8:**
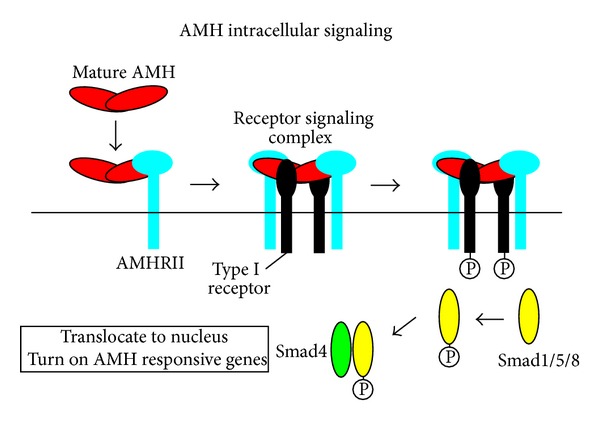
Signaling pathway of the AMH protein. Model showing how processing of AMH may regulate the assembly of the receptor signaling complex. Cleavage of full-length AMH results in a conformational change in the C-terminal domain, indicated by the shape and color change, which allows binding of AMRH-II. Binding of AMHRII induces dissociation of the proregion via a negative allosteric interaction between the receptor- and proregion-binding sites on the C-terminal dimer, indicated by the shape change. Results presented in this paper are consistent with proregion dissociation occurring before type I receptor engagement, but this has not been proven. Type I and II receptor-binding sites on the C-terminal dimer are indicated by either a I or a II; black labels indicate sites on the front of the dimer, and white labels indicate sites on the back of the dimer. From [2], Copyright The Endocrine Society, 2010, with permission.

Like other members of the TGF-ß family, AMH uses two types of membrane-bound serine/threonine kinase receptors for signal transduction. The AMH type II receptor, cloned in 1994 [62, 63], binds specifically to AMH and then recruits type I receptor, which phosphorylates intracytoplasmic proteins, the SMADs, allowing them to enter the nucleus to interact with target genes (Figure 8).

ALK2/ACVR1 [64, 65], ALK3/BMPR1A [66], and ALK6/BMPR1B [67], all type I receptors of the BMP family, have been found to interact with the AMH type II receptor. ALK2/ACVR1 [65] and ALK3/BMPR1A [66] have been shown to function redundantly in transducing AMH signal to provoke Müllerian duct regression. Conversely, ALK6/BMPR1B disruption does not affect Müllerian duct regression in male mice [64], and in the immature Sertoli cell line SMAT1 ALK6/BMPR1B inhibits AMH action [68].

Mutations of type II receptor, AMHR-II, are responsible for 48% of PMDS cases with documented genetic abnormalities (Figure 5). Clinical and biological features do not differ from those described above for AMH mutations, apart from the fact that serum AMH level is low/normal. AMH assay cannot discriminate between AMH and AMHR-II mutations in adulthood, because, in both instances, AMH levels are low. Even in childhood, a normal AMH level is not specific for AMHR-II mutations, since approximately 15% of PMDS cases are not associated with either AMH or AMHR-II mutations.

**Figure 9:**
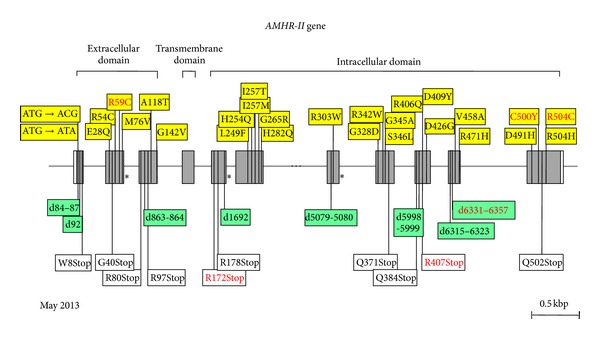
Mutations of *AMHR-II* gene in PMDS. Same representation as in Figure 6. All recurrent mutations are indicated in red. Asterisks represent splice mutations. Missense mutations are represented in yellow boxes, deletions (marked “d”) in green boxes, and nonsense mutations in white boxes. The deletion of 27 bases between bases 6331 and 6357 (d6331–6357 in exon 10) is extremely frequent: it is present in 21% of all PMDS families and in 44% of those with receptor mutations. Base numbering is from transcription initiation site, −78 bp from A of ATG.

The AMHR-II gene is composed of 11 exons, the first 3 coding for the receptor extracellular domain, exon 4 for most of the transmembrane domain, and the rest for the intracellular domain, where the kinase consensus elements are located. The gene has been mapped to the long arm of chromosome 12 [69]. The first AMHR-II mutation in PMDS, a splice mutation, was reported in 1995 [69]. Since then, 59 families, harboring a total of 49 abnormal AMHR-II alleles, have been studied in our laboratory, and an additional one has been reported in Boston (Figure 9) [70]. All exons except exon 4 may be affected. A 27-base deletion in exon 10 is present in approximately half the families with receptor mutations, nearly all of Northern European origin, suggesting a founder effect. Other recurrent mutations are much less frequent, apart from the nonsense R407Stop in exon 9, detected in 5 cases.

In approximately 15% of PMDS cases, all with a normal level of serum AMH, both the AMH and AMHR-II genes, including their proximal promoters and intronic sequences, are free of mutations. Several were born small and/or presented with various other congenital defects, such as jejunal atresia [71]. Mutations of the AMH type I receptors or cytoplasmic downstream effectors [72] are unlikely since these are shared with the BMPs and required for normal embryonic development. Inactivation [73] or dysregulation [74] of ß-catenin or dysfunction of other factors capable of interfering with AMH action might be involved.

## 5. Concluding Remarks

Assay of serum AMH now provides the pediatric endocrinologist with a new tool for investigating the function of the prepubertal testis, without the need for hCG stimulation. The assessment of both serum AMH, a marker of Sertoli cell function, and serum testosterone, reflecting Leydig cell function, is a simple and useful tool for the clinician. In DSD patients, when both hormones are below the normal male range, testicular dysgenesis should be suspected. AMH in the male range and low testosterone indicate Leydig cell-specific disorders. When both hormones are within or above the male range, androgen target organ defects are most likely. Finally, PMDS is a rare etiology of cryptorchidism in boys with virilized external genitalia: in these cases, low or undetectable serum AMH predicts mutations in the AMH gene while normal serum AMH drives attention to the AMHR-II gene. In boys with normally virilized genitalia, serum AMH helps in the assessment of the existence and function of testes. Undetectable AMH is indicative of anorchia, whereas low AMH indicates primary or central hypogonadism.

## Figures and Tables

**Table 1:** Etiopathogenic classification of disorders of sex development (DSD) in patients with a Y chromosome.

Etiopathogenic classification	Serum AMH	Serum T
(A) Malformative DSD		
Defective morphogenesis of the wolffian ducts		
Congenital absence of the vas deferens (Cystic Fibrosis)	Normal	Normal
Defective morphogenesis of the urogenital sinus and of the primordia of the external genitalia		
Cloacal malformations, aphallia, and isolated hypospadias	Normal	Normal

(B) Primary hypogonadism (early fetal-onset)		
(B.1) Dysgenetic DSD: whole testicular dysfunction		
Complete gonadal dysgenesis		
Y chromosome aberrations		
DSS duplications, 9p deletions (DMRT1/2?), 1p duplication (WNT4?)		
Gene mutations: SRY, CBX2, SF1, WT1, SOX9, DHH, MAMLD1, TSPYL1, DHCR7, and so forth	Undetectable	Undetectable
Partial gonadal dysgenesis		
Same as complete gonadal dysgenesis	Low	Low
Asymmetric gonadal differentiation		
45,X/46,XY, an other mosaicism, or Y chromosome aberrations	Low	Low
Ovotesticular gonadal differentiation		
46,XX/46,XY; an other mosaicism	Low	Low
(B.2) Nondysgenetic DSD: cell-specific dysfunction		
Leydig cell dysfunction		
Mutations in LH/CG-R, StAR, P450scc, P450c17, POR, cytochrome b5, 3*β*-HSD, and 17*β*-HSD	High in neonates and in pubertal age, normal in childhood	Low/undetectable
Sertoli cell dysfunction		
AMH gene mutations	Low/undetectable	Normal

(C) End-organ failure		
(C.1) Androgen end-organ failure		
Impaired DHT production		
5*α*-Reductase gene mutations	Normal	Normal
Androgen insensitivity syndrome (AIS)		
Androgen receptor mutations	Partial AIS: high in neonates, normal in childhood, and inadequately high at pubertal age Complete AIS: normal/low in neonates, normal in childhood, and very high at pubertal age	Normal/high
(C.2) AMH end-organ failure		
AMHR-II mutations	Normal	Normal

3*β*-HSD: 3*β*-hydroxysteroid dehydrogenase; 17*β*-HSD: 17*β*-hydroxysteroid dehydrogenase; AGD: asymmetric gonadal differentiation; AMH: Anti-Müllerian hormone; AMHR2: Anti-Müllerian hormone receptor type 2.

## Abbreviations

AIS: Androgen insensitivity syndrome
AMH: Anti-Müllerian hormone
AMHR-II: AMH receptor type 2
DSD: Disorders of sex development
hCG: Human chorionic gonadotropin
PMDS: Persistent Müllerian duct syndrome.
